# A Case of Phrenitis Resulting in Death, from the Inhalation of Sulphuric Ether

**Published:** 1845-07

**Authors:** J. Miller

**Affiliations:** Louisville, Ky.


ART. V.—*A Case of Phrenitis resulting in Death, from the Inhalation of Sulphuric Ether.* By Doct. J. MILLER, of Louisville, Ky.

It is not unknown to the profession that serious excitement, and even inflammation of the brain may result from the inhalation of the vapor of *Sulphuric ether;* but so far as the writer of this article is informed no fatal case of the kind has been brought before the public of late, in a way to call general attention to the danger of that practice, which is gaining a considerable prevalence in this country. Boys at school, and young people at social parties frequently amuse themselves by breathing the fumes of ether, which is easily done by saturating a handkerchief with that fluid, a lively intoxication, generally transient, being the consequence. It was at such a social meeting that the subject of the melancholy case, which I have been requested to report for the Western Journal, was induced to make the fatal experiment. In the hope of hearing her sing, her young friends pressed her to inhale ether, which others had done on the same evening without any unpleasant effects.

Miss E. A. was aged about 15 years, was of highly nervous temperament, of scrofulous diathesis, from an affection of which character she had suffered seriously in her childhood. At the time of breathing the ether she was in good health. Not being affected by the first trial with it she was prevailed upon to repeat it again and again, until she had made five successive trials. She then sat down apparently exhausted, in what was taken to be a pensive mood, and in that uncomfortable state soon left the party and returned home.

The next day found her with the symptoms of indisposition rather increased, and on the day following she complained of her head, had but little appetite, and was much debilitated. Up to this time, three days after inhaling the ether, she was able to go about, and on the third day, walked to church; but her giddiness and debility made it difficult for her to walk, and it was with a great effort that she was able to reach her house. On being questioned by her mother she represented that the breathing of the ether was attended with very painful sensations in the head, and produced a partial blindness, under which she had continued to labor ever since. By the evening of the third day she had grown quite ill; in attempting to walk up stairs she stopped suddenly, screamed so as to alarm the family, and complained of faintness and the pain in her head. Some aberration of mind was now manifest, and in the course of the night she became delirious, screaming, and evincing alarm at imaginary dangers. She spoke of the ether which she had inhaled as being the cause of her illness, declared in her lucid intervals that she had suffered ever since she breathed it, and cautioned those around her against its use. Under the remedies prescribed that night she appeared more comfortable the next morning, but during all that day she gave decided proofs of mental incoherence, which continued to increase. Her skin was hot. countenance flushed, pulse about 100, with much throbbing of the carotids; eyes half open and turned upwards, impaired vision and obtuse hearing. She complained of difficulty in turning her eyes downwards, but rolled them about in an unnatural way. When any subject was introduced she spoke rationally upon it for a moment, and then turned to some other, frequently to the experiment with the ether. Her nights became sleepless, and were spent in screaming, and loud talking upon all subjects, until she sunk finally into a comatose condition. Ten days after her indisposition commenced she had a slight spasm. Her pulse had reached 140, having gradually risen from day to day. On the twelfth day she expired. No autopsy was made.

Doubts were entertained by some, during the progress of this case, whether the symptoms were attributable to the inhalation of the ether; but no one, it is believed, doubted the existence of inflammation of the brain before all was over. To my mind it was clear enough, and the case ought to be a warning to the public. Parents should admonish their children of the danger, superintendents of schools should forbid the practice among their youth, and it would be well if apothecaries would decline selling the fluid when there was ground to suspect that it was intended for this purpose.

A day or two after writing the above, I learned accidenally, that Mr. Green had lost a son the same morning that the death of Miss A. occurred, and under similar circumstances. I immediately called upon the family, and was informed that this youth died under symptoms of mental derangement, but was unable to learn whether he had taken ether or not; he had returned home with the odor of it so strong about him as to attract attention, but no inquiry was made while living as to its use. Mrs. Green introduced her little daughter, aged 11 years, who at the time was laboring under some degree of mental alienation connected with a muscular disturbance, supposed to be chorea. On examination the following symptoms were observed: the countenance was wild, and a little vacant at intervals, sometimes indicating slight surprise; the eyes were full and somewhat suffused; the hearing was obtuse, and she complained of obscured vision, describing it as a mist, or thin cloud before the eyes. There was evident restlessness, mostly of the head and upper extremities; full and frequent inspirations were made; the mouth was often opened, and the head thrown back, as if to relieve slight stiffness of the muscles of the neck. The patient conversed incoherently at intervals, introducing strange topics, and had sometimes been observed to laugh immoderately without any assignable cause. She had been affected with these symptoms for several days before I saw her. When I visited her, she seemed capable of conversing on any subject introduced in so rational a manner, that the hullucination might have been overlooked by an uninterested observer. I noticed, however, that she conversed with clearness only so long as the topic was kept before the mind with some care; and this circumstance, together with the muscular disturbance, marked the case as similar to that of Miss A. I was informed that she had begun to show a greater inclination towards silence than formerly; her appetite was said to be good, and on a few occasions was thought to be inordinate. There was also evident difficulty in using the organs of speech; the pronunciation was indistinct and performed with some apparent effort. The subsultus mentioned above, simulated, it is true, that which obtains in chorea, but did not seem to me to be identical with it. The pulse was 98 in the minute, small and tense; the mouth was slightly dry, and the patient was observed to drink oftener than usual; she also complained of some pain in the head.

I could not ascertain, on inquiring of the patient whether she had taken ether or not; she thought she had not, but her father informed me two days afterwards, that he believed she had, and he has this morning stated to me that so deep is his conviction of the fact, not only that this case originated in that way, but also that his son was killed by the ether, that he has petitioned the City Council to interdict the practice of inhaling ether at the public schools. Before I called a second time at Mr. G.'s residence, his family had gone with the little girl to Lexington, in the hope of improving her health. Mr. G. has received a letter informing him that the patient was carried from the stage to her bed, and that what seemed only a trifling imperfection in her gait when she left home, has terminated in paralysis of the lower extremities, and that she is now helpless and speechless.

On reading a copy of my former letter, I find that I omitted to say, that subsultus obtained throughout the entire course of Miss A.'s case, from the time I first saw her until twelve hours before her dissolution.

Louisville, June 19, 1845.

[Professor Silliman, speaking of the relation of sulphuric ether to the animal economy, mentions that it is sometimes breathed for the sake of the sensations it produces, and adds that the practice is in every view improper, and may prove dangerous or even fatal. The late Dr. Godman has left on record the particulars of a case, in which the effects of inhaling the vapor of ether were eminently serious. A female, predisposed to consumption, after breathing it for its exhilarating effects, suffered with cough, derangement of mind, and pain; had several attacks of violent syncope, and remained ill for some time.—(Western Reporter, vol 2, p. 111). Professor T. D. Mitchell, in his Chemistry, refers to the amusement of inhaling ether, and says that, some years ago in Philadelphia, several cases occurred in which "delirium, and even phrenitis was induced" by it, some of "which ended fatally." Pereira also speaks of its occasionally deleterious effects when inhaled. "If the air be too strongly impregnated with ether,"

he observes, "stupefaction ensues. In one case this state continued with occasional periods of intermission for more than thirty hours: for many days the pulse was so much lowered that considerable fears were entertained for the safety of the patient. In another case, an apoplectic condition, which continued for some hours, was produced."

Although, as remarked by the last writer, the effects of the vapor of ether upon the system are analogous to those caused by the exhilarating gas, there can be no question that they are far from being as harmless. In our reading we have met with no case, in which serious effects followed breathing the protoxide of nitrogen. Professor Silliman has never seen this gas do mischief. Pereira speaks of having administered it to about a hundred persons, and does not refer to any injurious consequences. We have administered it to nearly two hundred young men, in the last thirteen years, and have not yet witnessed a case in which its influence was more than temporary. Even headache is not a common consequence of respiring it. But it is very different with the vapor of sulphuric ether. We never administered it, or saw any one under its influence; but we have been assured by those who have inspired it, that it is generally followed by pain in the head, sometimes by pain in the chest, threatening inflammation, and that the cerebral excitement, bordering upon delirium, often persists for hours. From the history of the above cases by Dr. Miller, we do not hesitate to believe that the young lady died from the effects of breathing ether.

